# Rapid and Reversible Impairments of Short- and Long-Term Social Recognition Memory Are Caused by Acute Isolation of Adult Rats via Distinct Mechanisms

**DOI:** 10.1371/journal.pone.0065085

**Published:** 2013-05-31

**Authors:** Hadar Shahar-Gold, Rotem Gur, Shlomo Wagner

**Affiliations:** Sagol Department of Neurobiology, University of Haifa, Haifa, Israel; University of Queensland, Australia

## Abstract

Mammalian social organizations require the ability to recognize and remember individual conspecifics. This social recognition memory (SRM) can be examined in rodents using their innate tendency to investigate novel conspecifics more persistently than familiar ones. Here we used the SRM paradigm to examine the influence of housing conditions on the social memory of adult rats. We found that acute social isolation caused within few days a significant impairment in acquisition of short-term SRM of male and female rats. Moreover, SRM consolidation into long-term memory was blocked following only one day of social isolation. Both impairments were reversible, but with different time courses. Furthermore, only the impairment in SRM consolidation was reversed by systemic administration of arginine-vasopressin (AVP). In contrast to SRM, object recognition memory was not affected by social isolation. We conclude that acute social isolation rapidly induces reversible changes in the brain neuronal and molecular mechanisms underlying SRM, which hamper its acquisition and completely block its consolidation. These changes occur via distinct, AVP sensitive and insensitive mechanisms. Thus, acute social isolation of rats swiftly causes changes in their brain and interferes with their normal social behavior.

## Introduction

Social species, by definition, form social organizations that have co-evolved with the behavioral, neural, hormonal, cellular, and genetic mechanisms supporting them [1], [2]. In humans, creating and maintaining social relationships are among our most common and fundamental activities, yet little is known about the brain mechanisms involved. Social isolation has significant negative effects on human health [3] and is as strong a risk factor for morbidity and mortality as smoking, obesity and high blood pressure [4], [5]. Social isolation also predicts cognitive decline and risk for Alzheimer’s disease [6]. However, the biological mechanisms underlying the effects of social isolation on human cognition are still elusive.

Mammalian social organizations require the ability of an individual to recognize and remember other individuals of the same species (conspecifics). This social recognition memory (SRM) can be examined in rats or mice using their innate tendency to investigate novel conspecifics more persistently than familiar ones [7]. SRM is quantitatively assessed by the reduction in time an individual spends investigating an individual in their second encounter, relative to their first one [8]. Using this method.

SRM was intensively investigated in rats and mice during the last three decades. Yet, the ability of these animals to acquire long-term SRM remains questionable: while only a short-term (<120 min) SRM was reported for male rats [9], [10], [11], [12], mice were shown to retain an SRM for more than a week if housed in groups rather than in solitary cages [13]. Interestingly, even rats showed acquisition of long-term SRM following pharmacological treatments, such as administration of arginine-vasopressin (AVP) [14], [15], [16], [17], [18]. Indeed, this neuropeptide, together with the similar peptide oxytocin (OXT), have been found to be crucial to SRM acquisition in rats and mice [19], [20].

Here we show for the first time that, if kept in group housing, both male and female adult rats can acquire long-term SRM. In contrast, acute social isolation causes a rapid impairment in both short- and long-term SRM. In further experiments with adult males, we show that both impairments are reversible, but with different time courses. Moreover, only the impairment in long-term SRM is reversed by systemic AVP injection, suggesting distinct mechanisms for isolation-induced impairment of short- and long-term SRM.

## Materials and Methods

### Ethics Statement

All experimental protocols were approved by the Animal Care and Use Committee of the University of Haifa.

### Animals

Subjects were adult Wistar-Hola males (7–8 wk, 250–300 g) or female (7–8 wk, 150–200 g) rats or adult Sprague-Dawley (SD) male rats (300 g, n = 27). These were kept in 12 h light/dark cycle, 22±2°C, food and water available *ad libidum* under veterinary supervision. Rats used as social stimuli were juveniles (30 g) of different strains (SD or Wistar Hannover/Hola).

### Social Discrimination

The SD rats were housed in groups of 2–5 animals per cage (60×40×20 cm) and handled daily for 2 weeks before testing. The Wistar Hannover/Hola juveniles were held in groups of 2 of the same strain and were used as social stimuli in random order. Following 30 min habituation in the experimental cage (60×40×20 cm), the adult subject was unlimitedly exposed to a juvenile for 1 h. A day later the adult subject was exposed simultaneously to the same juvenile and to a novel juvenile of the other strain, each confined to a transparent plastic corral (9 cm in diameter) slotted such that the adult’s nose could touch the juvenile’s body (each slot1×13 cm, 5 slots per side). The duration of investigatory behavior of the adult towards each juvenile was blindly measured with a stopwatch, once during the experiment and twice later from a video recording of the experiment, each time by a different observer. The final investigation time represents an average of all three measurements.

### Social Recognition

For this and all following experiments subjects were adult Wistar-Hola male or female rats. Normally, the animals were housed in groups of five per cage (60×40×20 cm). For the social isolation condition, animals were housed with no handling in solitary cages (50×30×20 cm) for 7–14 days, if not otherwise specified. Solitary and group cages were kept in the same room. Juvenile (3 wk, 30–35 g) male Wistar-Hanover or SD rats were used as social stimuli. Whenever two distinct social stimuli were used in the same test, they were of different strains in a random order. All experiments were blindly performed by two trained graduate students and started with a 1 h habituation of the adult rat to a fresh cage (50×30×20 cm) in dim light. All encounters lasted for a fixed duration (2 or 5 min), at the end of which the juvenile stimulus was returned to its original cage and could have been immediately used for a new test. The duration of social investigation, including any contact between the subject’s nose and the juvenile’s body, following behavior or any investigatory behavior directed towards the stimulus animal that does not involve direct contact between the animas was measured using a stopwatch. Using this methodology we have performed the following experiments:

- **Social Recognition Memory (SRM).** An encounter with a novel juvenile for 2 or 5 min followed by a second encounter 30, 60, 120, or 180 min later. In most cases, a third encounter with a novel juvenile of a different stain followed the second one by 30 min, to ensure stimulus specific memory.
- **Retroactive facilitation.** Three consecutive 5 min encounters with the same juvenile, separated by 10 min. 1 or 7 days later the subject was exposed to the same juvenile for 5 min, followed 30 min later by an encounter with a novel juvenile.
- **Object Recognition Memory.** Same as SRM but with object stimuli (paper cup, plastic Lego cube, glass bottle).
- **AVP administration.** [Arg^8^]-Vasopressin acetate salt (Sigma) was dissolved in 0.1 M acetic acid to a concentration of 1 µg/µl and stored at −80°C in 20 µl aliquots. On the day of use, each aliquot was diluted in 5 ml saline solution (0.9% NaCl). One minute after the first encounter with the juvenile each rat subject received a subcutaneous injection of saline or 6 µg/kg AVP in random order. Tests with saline or drug administration in the same rat were always separated by at least 48 h.

### Statistical Analysis

All statistical analyses were carried out with SPSS 19.0 software for Windows. We used parametric t-test and ANOVA only if data were found to be normally distributed, otherwise non-parametric tests were used. SRM was confirmed if a significant difference (*p*<0.05, two tailed paired t-test) was found between the familiar and novel juveniles. We compared two encounters of the subject with the same juvenile (A1 and A2) only if separated by ≤180 min.

## Results

### Long-term SRM in Adult Male Rats

**Figure 1:**
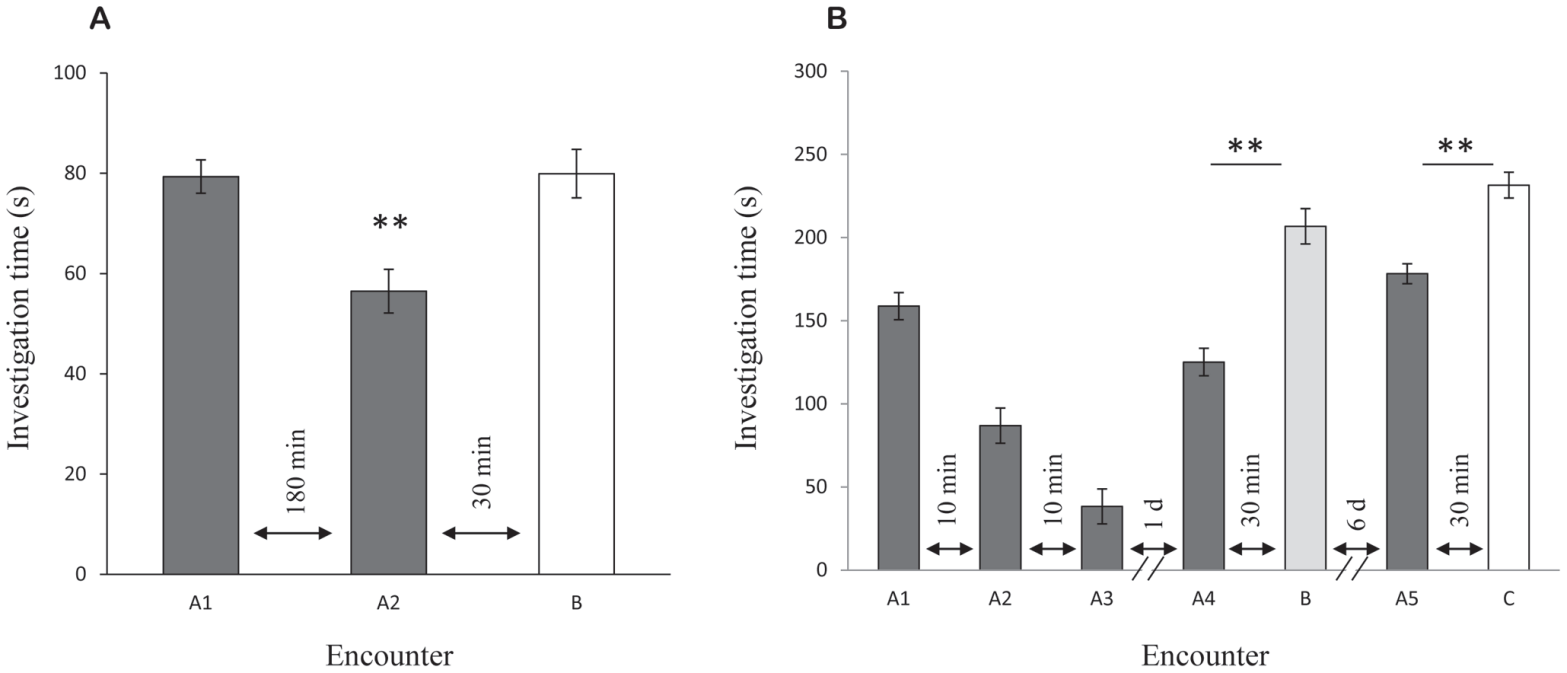
Adult male rats acquired long-term SRM (mean±SEM): Adult male rats (n = 18) showed a significant reduction in social investigation time (SIT) during the second encounter with a juvenile (A2) 180 min after the first encounter with the same animal (A1). This SIT was also less than during an encounter with a novel juvenile (B) 30 min later. All encounters lasted 2 min. No significant difference was found between the two encounters with a novel juvenile (A1,B). Repeated ANOVA: F _(2,34)_ = 22.72, *p*<0.001, Bonferroni *post hoc*: ** *p*<0.01. A) After three consecutive 5 min encounters with the same juvenile (A1–3), adult male rats (n = 8) retained long-term SRM, assessed by the significant difference in SIT between the encounters with this juvenile and novel one (1 d - A4 vs. B; 7 d - A5 vs. C) 30 min later. Paired t-test: ** *p*<0.01.

To determine whether male rats can acquire long-term SRM each subject experienced two 2 min encounters 180 min apart with the same juvenile (A1,A2), followed 30 min later by an encounter (B) with a novel juvenile (Fig. 1A). The mean social investigation time (SIT) decreased significantly by ∼30% between the two encounters with the same juvenile, while the SIT in the third encounter increased to a level not significantly different from the first encounter (repeated ANOVA, F_(2,34)_ = 22.72, *p*<0.01). Thus, adult male rats retain SRM for more than 180 min.

Next, we examined if this memory is preserved for more than several hours. The experiment was as described above (Fig. 1A) but with a 1 d interval between the first and second encounters with the same juvenile. Since these two encounters occurred on different days, they were not compared with each other. Instead, we compared the SITs of the encounter with the familiar juvenile (A2) and the encounter with a novel juvenile 30 min later (B). We found that the familiar juvenile was investigated for ∼15% less time than the novel one (75.6±14.6 s and 91.0±19.9 s, respectively), a difference which was statistically significant (paired t-test t_(13)_ = −4.77, *p*<0.01). This difference suggests SRM preservation for at least 24 h following a single 2 min encounter. To reject the possibility that such a difference may be found between two novel stimuli, we compared the SIT between two 2 min encounters, 30 min apart, each with a novel juvenile and found no significant difference between them (80.4±20.8 s and 89.1±13.2 s; paired t-test t_(4)_ = −1.74, *p*>0.05).

To extend our results for longer periods we used retroactive facilitation [18] to enhance SRM acquisition. The subject encountered the same juvenile in three consecutive 5 min encounters 10 min apart. Long-term SRM was examined 1 d and 7 d later. As shown in Fig. 1B, the SIT gradually decreased between the three consecutive encounters with the same juvenile (A1–3) (paired t-test − A1 vs. A3: t_(7)_ = 10.28, *p*<0.01). A day later, the same juvenile (A4), was investigated for only ∼60% of the time spent with a novel juvenile (B) (A4 vs. B t_(7)_ = −6.67, *p*<0.01). When this procedure was repeated 6 d later, we again found a significant difference in SIT between the two juveniles; the familiar juvenile (A5) was investigated for ∼25% less time than the novel one (C) (A5 vs. C t_(7)_ = −5.75, *p*<0.01). Thus, adult male rats can retain SRM for more than a week. It should be noted that the ratio between the SITs of the familiar and novel juveniles (RDI) declined significantly between 1 d and 7 d (t -test: t_(7)_ = −2.85; *p*<0.05), suggesting SRM deterioration over time.

To ensure that this long-term SRM was not dependent on strain or the experimental paradigm, we examined long-term social memory in Sprague-Dawley (SD) rats using the social discrimination paradigm. On the day after a 1 h exposure to a novel juvenile, adult rats exposed simultaneously to both the familiar and to a novel juvenile, investigated the familiar juvenile for only ∼60% of the time they investigated the novel one (61.7±23.8 s and 98.15±27.3 s, respectively). The differences in investigation time between the two juveniles were statistically significant (paired samples Wilcoxon signed-rank test, *p*<0.001), confirming long-term SRM as independent of stimulus paradigm or rat strain. Our adult male rats acquired long-term SRM and retained it for at least a week, contrasting with the many reports that adult male rats do not retain SRM for more than 1 h [9], [10], [11], [12]. We noted that mice show long-term SRM if kept in groups, while socially isolated mice display only short-term SRM [13]. Our experiments were performed with rats in group housing, but almost all previous studies have used socially isolated rats. Thus, the different housing conditions may explain the discrepancy. To investigate this possibility we next examined the effect of housing conditions on long-term SRM.

### Social Isolation Specifically Impaired Long-term SRM

**Figure 2:**
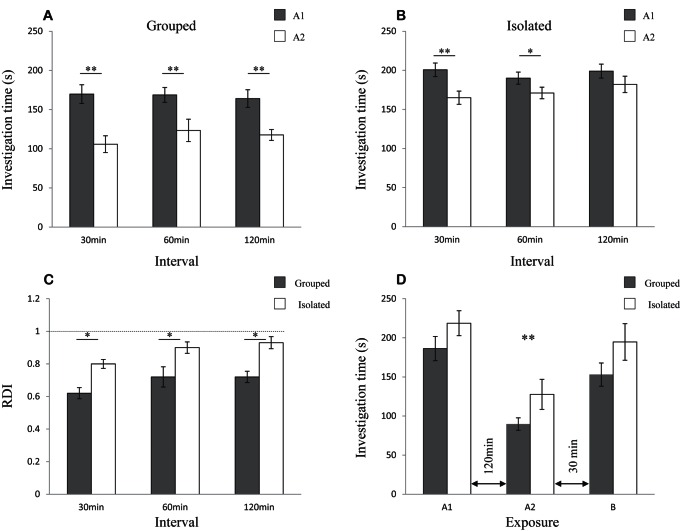
Housing conditions specifically impaired long-term SRM (mean±SEM): A) Rats in group housing displayed a significant decrease in SIT during the second 5 min encounter (A2, empty bars) compared to the first (A1, gray bars), regardless of the time interval between the encounters. Paired t-test: ** *p*<0.01, n = 10 per group. B) Socially isolated rats displayed a significant decrease in SIT measured during the second encounter only for intervals of 30 and 60 min but not for a 120 min interval. Paired t-test: ** *p*<0.01, * *p*<0.05, n = 10 per group. C) Isolated rats (empty bars) showed a significantly higher relative duration of investigation (RDI) than animals from group housing (gray bars) at all three time intervals. t-test: * *p*<0.05, n = 10 per group. D) Rats from group housing (gray bars, n* = *10) and socially isolated rats (empty bars, n = 10) similarly retained object recognition memory for 120 min following a single 5 min exposure. Both groups showed a significant decrease in investigation time during the second exposure to an object (A2), compared with the first exposure (A1) 120 min earlier and compared with exposure to a novel object (B) 30 min later. Repeated ANOVA, mixed model, F_(2,36)_ = 26.1, *p*<0.001, no interactions (*p*>0.05), Bonferroni *post hoc*: ** *p*<0.01 between exposures, no difference between groups (*p*>0.05).

In order to compared the SRM between group-housed and socially isolated rats, we exposed each subject to two 5 min encounters with the same juvenile separated by 30, 60, or 120 min. Rats from group housing displayed SRM at all intervals according to our t-test criterion (Fig. 2A, paired t-test: 30 min t_(9)_ = 8.19, *p*<0.01; 60 min t_(9)_ = 4.48, *p*<0.01; 120 min t_(9)_ = 6.7, *p*<0.01). However, isolated rats retained SRM for 30 and 60 min but not for 120 min (Fig. 2B, paired t-test: 30 min t_(9)_ = 7.35, *p*<0.01; 60 min t_(9)_ = 2.67, *p*<0.05; 120 min t_(9)_ = 2.19, *p*>0.05). A statistically significant difference between the housing conditions was found in the RDI calculated for the two encounters at all intervals (Fig. 2C, t-test: 30 min t_(18)_ = −4.672, *p*<0.01; 60 min t_(18)_ = −2.5, *p*<0.05; 120 min t_(18)_ = −3.97, *p*<0.01). Thus, as compared to group-housed rats, SRM acquired by isolated rats is significantly weaker and is retained for a much shorter time, less than 120 min.

It should be noted that the rats in group housing showed ∼15% lower SIT values during the first encounter with a novel juvenile (167.6±24.9 s) than isolated rats (196.7±18.6 s). This difference was statistically significant (t-test: t_(18)_ = −2.97, *p*<0.01).

To determine whether the isolation-induced impairment was specific for social memory, we tested group-housed and socially isolated rats in the object recognition paradigm with 120 min interval. Both subject groups showed a significant reduction in investigation time between the first and second exposures to the same object (Fig. 2D), with no significant difference between the two groups (repeated ANOVA, mixed model, F_(2,36)_ = 26.1, *p*<0.001, no interactions *p*>0.05 Bonferroni *post hoc*). Thus, it is specifically the social memory that is impaired in isolated rats. These results are in accordance with a study showing that the impairment induced in adult male mice by social isolation was specific to SRM and did not affect other learning paradigms such as object recognition or inhibitory avoidance [21]. Yet, in mice long-term SRM was reported to be impaired already 1 d following social isolation [13]. Therefore, we next examined the time course of the social-isolation induced SRM impairment in adult male rats.

### The Impairment of Long-term SRM Induced by Isolation was Reversible

**Figure 3:**
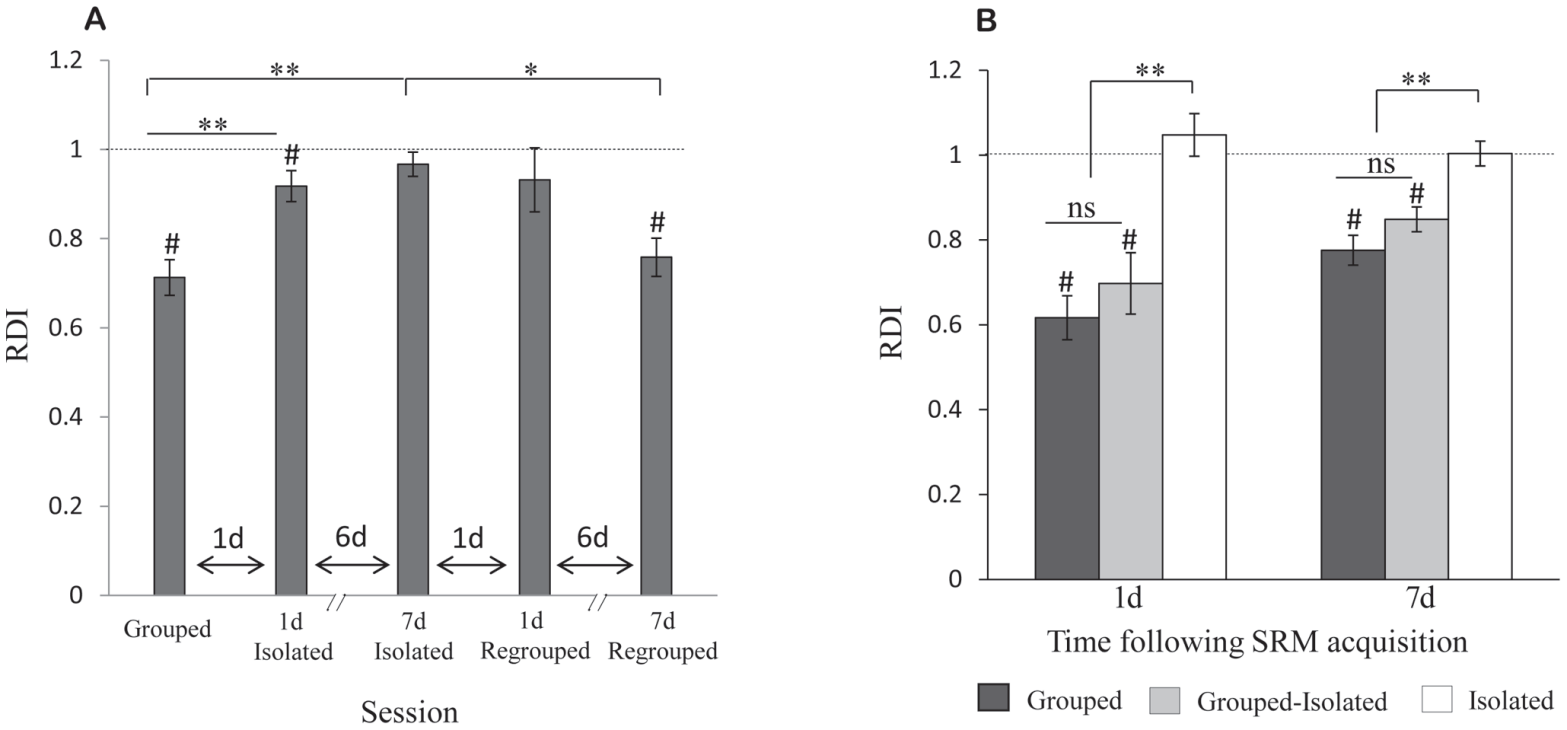
Social isolation rapidly induced a reversible impairment in consolidation of long-term SRM (mean±SEM). A) RDI values of two 5 min encounters, 120 min apart, with the same juvenile. This test was repeated with the same group of adult animals (n = 13) while their housing conditions were changed, using new stimuli for every session. Long-term SRM was confirmed for every session if a paired t-test found a significant reduction in SIT between the first and second (**#** *p*<0.05). After isolation for 1 day (1 d isolated) the rats showed a significant increase in their RDI values from when they were housed in groups (Grouped) and following 7 days of isolation (7 d isolated) the rats were unable to acquire long-term SRM. This effect was reversed following 7 days (7 d Regrouped), but not 1 day (1 d Regrouped) of return to group housing. Repeated ANOVA, F_(4,48)_ = 6.96, *p*<0.001, Bonferroni *post hoc*: *p<0.05, **p<0.01. B) RDI values of 5 min encounters with the familiar and a novel juvenile 30 min apart, 1 d (left) and 7 d (right) after three consecutive 5 min encounters with the familiar juvenile (retroactive facilitation, Fig. 1B). Male rats from group housing, isolated immediately after the retroactive facilitation (Grouped-Isolated, light gray bars), displayed SRM both 1 day and 7 days later, as did males kept in group housing throughout the experiment (Grouped, dark gray bars). In contrast, rats kept in solitary cages for 7 days before SRM acquisition and throughout the experiment (Isolated, empty bars) did not retain the memory even for 1 day. Long-term SRM was assessed by paired t-test (# *p*<0.05) on SIT values between the encounters with the familiar and the novel juvenile. For both 1 day and 7 days intervals no statistically significant difference in RDI was found between Grouped and Grouped-Isolated animals, whereas Isolated rats differed from the other two groups (1-way ANOVA, 1 d: F_(2,21)_ = 14.9 *p* = 0.001; 7 d: F_(2,21)_ = 13.7, *p*<0.001, Bonferroni *post hoc*: **p<0.01, n = 8 per group.

To determine the time course of the impairment in long-term SRM, two 5 min encounters with the same juvenile separated by 120 min tested for long-term SRM. This test was repeated with the same group of adult males (n = 13) using novel stimuli for every test, while the housing conditions were changed during the course of the experiment. As shown in Fig. 3A, group-housed rats (Grouped) showed a normal SRM, reflected by the statistically significant reduction in SIT between the two encounters (paired t- test: t_(12)_ = 6.52, *p*<0.01). Immediately after this session the rats were placed in solitary cages and the experiment was repeated 1 d later (1 d Isolated). At this stage there was a pronounced reduction in the RDI value, although a weak memory was still preserved (t_(12)_ = 2.45, *p*<0.05). Six days later no SRM was shown by the isolated rats (7 d Isolated) (t_(12)_ = 1.42, *p*>0.05). The rats were then returned to group housing and the experiment repeated 1 d later (1 d Regrouped), with no SRM displayed (t_(12)_ = 1.58, *p*>0.05). When examined 6 d later (7 d Regrouped), these rats did show long-term SRM (t_(12)_ = 5.27, *p*<0.01) and RDI values were significantly reduced compared to the 7 d Isolated condition, but not the Grouped condition (repeated ANOVA, F_(4,48)_ = 6.96, *p*<0.001, Bonferroni *post hoc p*<0.01). We draw three conclusions from this experiment: 1, long-term SRM was severely impaired after even one day in social isolation. 2, this impairment was reversible following regrouping of the isolated animals. 3, the reversal of the impairment was slower than its induction.

### Social Isolation Impaired Consolidation of SRM

Social isolation may impair either the consolidation of long-term SRM or its recall. To distinguish between these possibilities we performed the same experiment as in Fig. 1B, with three groups of animals: one group (Grouped) was held in group housing throughout the experiment. The second group (Isolated) was housed in solitary cages for seven days before the experiment and throughout it. The third group (Grouped-Isolated) was placed in solitary cages only after acquiring the memory by retroactive facilitation. Both Grouped and Grouped-Isolated rats showed SRM 1 d and 7 d after memory acquisition (paired t-test − Grouped: 1 d t_(7)_ = −6.67, *p*<0.01; 7 d t_(7)_ = −5.75, *p*<0.01; Grouped-Isolated: 1 d t_(7)_ = −4.63, *p*<0.01; 7 d t_(7)_ = −5.05, *p*<0.01) with no significant difference between them (Fig. 3B, one-way ANOVA - 1 d: F_(2,21)_ = 14.9, *p<*0.01; 7 d: F_(2,21)_ = 13.7, *p*<0.01, Bonferroni *post hoc p<0.01*). In contrast, isolated rats showed no SRM at both intervals. Since even seven days of isolation did not impair the recall of memory acquired during group housing, we conclude that social isolation interfered with the consolidation of long-term SRM, but not with the recall of a memory previously acquired in group-housing condition.

### Short-term SRM was also Impaired by Social Isolation

Previous studies in rats reported that the minimal exposure time needed for SRM was 150 s [11]. Yet, our experiments revealed that, a 2 min exposure was sufficient to induce long-term SRM (Fig. 1A). As previous studies have mostly used isolated rats, we next investigated whether isolated rats are impaired, not only in the consolidation of long-term SRM, but also in the minimal exposure time required for SRM acquisition. We thus compared the SRM of group-housed and socially isolated rats after a 2 min encounter with a novel juvenile.

**Figure 4:**
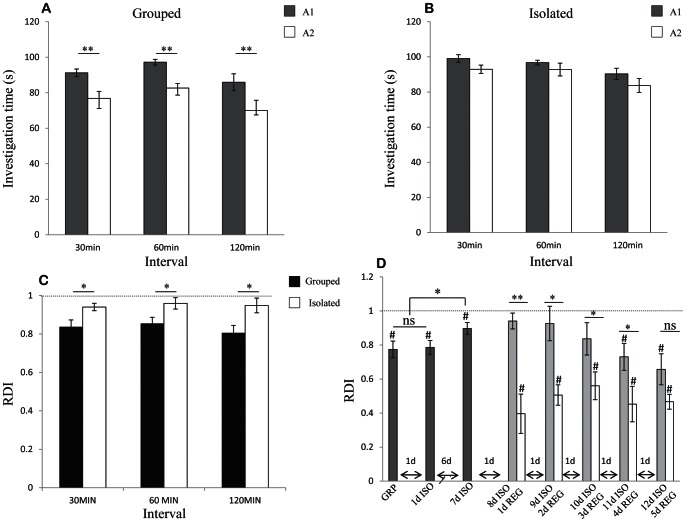
Short-term SRM was also impaired by social isolation (mean±SEM). A) Rats in group housing displayed a significant decrease in SIT during the second 2 min encounter with a juvenile (A2, empty bars) compared to the first (A1, gray bars), regardless of whether the encounters were separated by 30, 60 or 120 min. Paired t-test: ** *p*<0.01, n = 10 for 30 and 120 min, n = 9 for 60 min. B) Socially isolated rats did not display a significant decrease in SIT during the second 2 min encounter regardless of the interval between the encounters. Paired t-test: *p*>0.05, n = 10 for 30 and 60 min, n = 12 for 120 min. C) Socially isolated rats (empty bars) showed a significantly higher RDI than animals from group housing (black bars) at all three time intervals. Paired t-test: * *p*<0.05, Grouped: n = 10 for and 120 min, n = 9 for 60 min. Isolated: n = 10 for 30 and 60 min, n = 12 for 120 min. D) RDI values for two 2 min encounters with the same juvenile 30 min apart. This test was repeated with the same group of adult males (n = 20) while housing conditions were changed, using new stimuli for every session. Short-term SRM was confirmed for every session if a significant reduction in SIT between the first and second encounters was found using paired t-test (# *p*<0.05). Dark grey bars represent RDI values of 20 adult male rats in group housing (GRP) as well as after 1 day (1 d ISO) and 7 days (7 d ISO) of social isolation. Note that, unlike long-term SRM (Fig. 3A), there was no significant increase in RDI compared to animals from group housing (GRP) after 1 day of social isolation. These animals were then divided into two groups: one group remained in social isolation (ISO, light-grey bars) and the other was returned to group housing (REG, empty bars). Both groups performed the same test on a daily basis. A profound difference between the two groups appeared even 1 day following division (t-test *p<0.05, ** *p*<0.01), but was gradually reduced. On the last day of the experiment animals in both groups showed short-term SRM, with no significant difference between them.

As shown in Fig. 4A, group-housed rats showed a relatively constant and statistically significant reduction of ∼15% in SIT between the first and second encounters with the same juvenile separated by 30, 60 or 120 minutes (paired t-test − 30 min: t_(9)_ = 4.82, *p*<0.01; 60 min: t_(8)_ = 4.58, *p*<0.01; 120 min: t_(9)_ = 6.18, *p*<0.01). In contrast, rats isolated for a week (Fig. 4B) did not show any significant decrease in SIT between the two encounters (30 min: t_(9)_ = 1.45, *p*>0.05; 60 min: t_(9)_ = 1.35, *p*>0.05; 120 min: t_(11)_ = 1.5, *p*>0.05), even with only 30 min between them. Accordingly, group-housed rats showed significantly lower RDI values than socially isolated rats (Fig. 4C, t-test − 30 min: t_(18)_ = 2.78, *p*<0.05; 60 min: t_(17)_ = 2.35, *p*<0.05; 120 min: t_(20)_ = 2.6, *p*<0.05). We conclude that socially isolated rats were indeed impaired in short-term SRM and needed an exposure time of more than 120 s to acquire it. Yet, unlike long-term SRM, short-term SRM was reported to be unimpaired following social isolation of mice [13]. This difference between the impairments in long- and short-term SRM suggest that the may involve distinct mechanisms. To further explore this possibility we next investigated the time course of the social-isolation induced impairment in short-term SRM.

### The Time Course of the Impairment in Short-term SRM Induced by Social Isolation

To examine the time course of the impairment due to social isolation in short-term SRM, we used two 2 min encounters with the same juvenile separated by 30 min. This test was repeated with the same group of adult animals (n = 20), using different juveniles for every test, while the housing conditions were changed during the course of the experiment (Fig. 4D). Fitting the previous results (Fig. 4A), group-housed rats showed significantly reduced investigation time in the second encounters with the same juvenile (∼20%), confirming acquisition of short-term SRM (Fig. 4D, GRP, paired t-test: t_(19)_ = 4.89, *p*<0.01). The animals were then placed in solitary cages and examined a day later (1 d ISO) using the same test, with no significant change observed in SRM acquisition (t_(19)_ = 5.41, *p*<0.01). However, when the same animals were examined after seven days in social isolation (7 d ISO), there was a significant increase in RDI values, suggesting impaired short-term SRM (one way ANOVA, (F_(2,38)_ = 3.434, *p*<0.01; Bonferroni *Post hoc p*<0.01). Next, half the animals were returned to group housing (REG, empty bars) while the other half remained in isolation (ISO, gray bars), and both groups were examined with the same test every day for 5 days. Figure 4D shows that one day after regrouping (1 d REG), the rats showed a strong and significant reduction of ∼60% in SIT between the first and second encounters with the same juvenile, demonstrating a profound acquisition of short-term SRM (paired t-test: t_(9)_ = 5.24, *p*>0.01). In contrast, the rats left in solitary cages (8 d ISO) showed no short-term SRM (t_(9)_ = 1.38, *p*>0.05). The significant difference in RDI values between the isolated and regrouped animals at this stage of the experiment (t-test − 8 d ISO vs. 1 d REG: t_(18)_ = 4.37, *p*<0.01) showed that even a single day in different housing conditions was enough to cause a profound change in short-term SRM. A similar difference was observed one and two days later (9 d ISO vs. 2 d REG: t_(18)_ = 2.29, *p*<0.05 and 10 d ISO vs. 3 d REG: t_(18)_ = 2.2, *p*<0.05, respectively). As this test continued on a daily basis, the socially isolated animals started to show decreasing RDI values, until on the last day of the experiment there was no significant difference between the groups (11 d ISO vs. 4 d REG: t _(18)_ = 2.12, *p*<0.05; 12 d ISO vs. 5 d REG: t _(18)_ = 1.88, *p>*0.05).

Thus, the impairment in short-term SRM induced by isolation is reversible, similarly to long-term SRM. However, the time course of the two impairments differed. While the impairment in long-term SRM was fully induced within 1 d of isolation and took more than 1 d to be reversed, the impairment in short-term SRM was induced more slowly (>1 d) but was reversed within 1 d of regrouping. The experiment also showed that the rats did not require many social interactions for intact SRM. As shown in Fig. 4D, even two 2 min encounters per day for several days fully reversed the effect of social isolation on short-term SRM.

The different time courses of induction and reversal of the impairments in long- and short-term SRM suggest that they are caused by different mechanisms. To further explore this possibility we next investigated the effect of systemic AVP administration on these impairments, as AVP infusion was previously shown to allow long-term SRM acquisition even in socially isolated rats [14], [17], [22], [23].

### The Impairments in Short- and Long-term SRM were Differentially Affected by AVP

**Figure 5:**
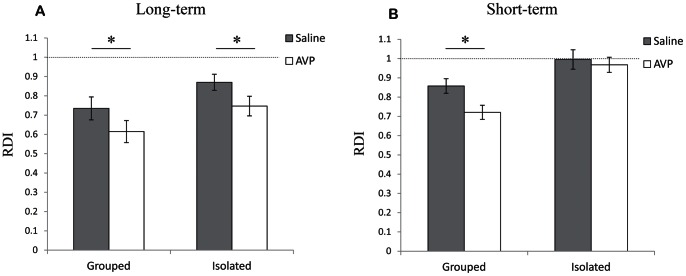
Arginine-vasopressin (AVP) administration affected the impairment induced by social isolation in long-, but not in short-term SRM (mean±SEM). A) RDI values of two 5 min encounters with the same juvenile 120 min apart. Male rats from group housing (left, n = 11) and socially isolated rats (right, n = 20) were given either saline (grey bars) or AVP (empty bars) subcutaneously 1 min after the first encounter. In both housing conditions AVP administration significantly reduced the RDI compared to saline administration, showing SRM improvement. Paired t-test: * *p*<0.05. B) RDI values of two 2 min encounters with the same juvenile 60 min apart. Male rats from group housing (left, n = 7) and socially isolated rats (right, n = 8) received either saline (grey bars) or AVP (empty bars) subcutaneously as above. In contrast to long-term SRM (a), AVP administration significantly improved short-term SRM compared to saline administration only in rats from group housing. No improvement was observed in socially isolated animals. Paired t-test: * *p*<0.05.

We examined the effect of systemic AVP administration on short- and long-term SRM by injecting AVP subcutaneously 1 min after an encounter with a novel juvenile. This experiment was carried out with group-housed and socially isolated animals, using either the short- (2 min encounters, 60 min interval) or long- (5 min encounters, 120 min intervals) term paradigms. As shown in Fig. 5A–B, AVP injections in group-housed animals caused a significant improvement in both the long- and short-term memory, compared to saline injections (1- tail paired t-test − long-term SRM: t_(10)_ = 1.84, *p*<0.05; Short term SRM: t_(6)_ = 2.33, *p*<0.05). In isolated animals, however, AVP injections caused a significant improvement in long-term (t_(19)_ = 1.98, *p*<0.05), but not in short-term SRM (t_(7)_ = 0.382, *p*>0.05). Since the impairments induced by social isolation in long- and short-term SRM not only differ in their time course but are also differentially affected by AVP administration, we conclude that they are mediated by distinct mechanisms.

### SRM of Female Rats was also Sensitive to Social Isolation

**Figure 6:**
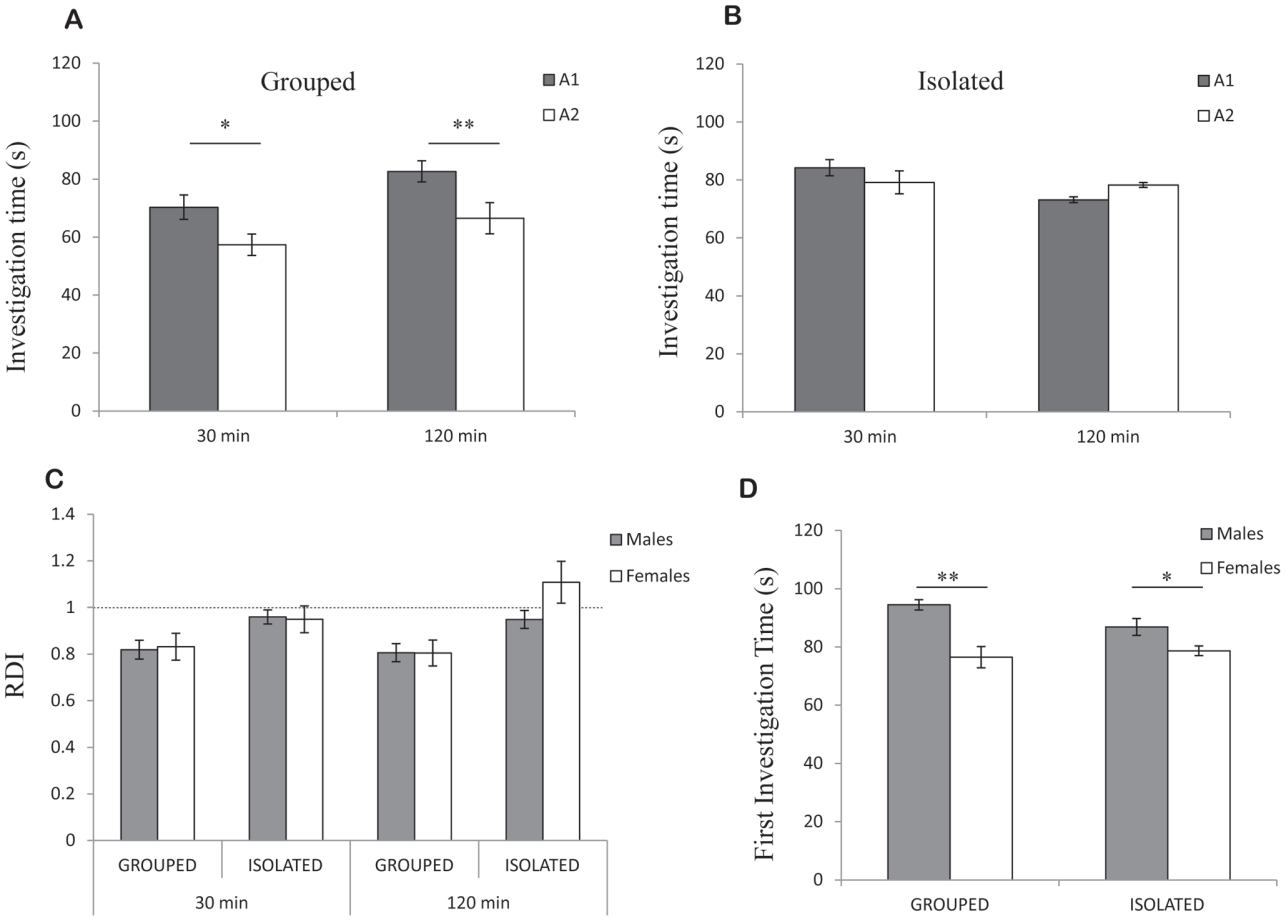
Housing conditions affected acquisition of SRM in female rats (mean±SEM): A) Females from group housing displayed a significant decrease in the SIT during the second 2 min encounter (A2, empty bars) compared to the first (A1, gray bars) when encounters were separated by 30 or 120 min. Paired t-test: * *p*<0.05, **p<0.01, n = 10 per group. B) Socially isolated females displayed no significant decrease in SIT during the second 2 min encounter. Paired t-test: *p*>0.05, n = 10 per group. C) RDI values of males vs. females in different housing conditions and different intervals between encounters. No significant difference was found (t-test) between males and females SRM. n = 10 per group. D) First investigation time of grouped and isolated males vs. females. In both housing conditions females investigated the social stimuli at the first encounter for significantly less time than males. t-test: * *p*<0.05, ** *p*<0.01, n = 10 per group.

Female rats were previously reported to retain SRM for longer periods than males [24], suggesting that their social memory is less sensitive to the effect of social isolation. To investigate this possibility, we compared the SIT values of two 2 min encounters with the same juvenile, separated by either 30 or 120 min, between group-housed and socially isolated female rats. As shown in Fig. 6A, for both intervals female rats from group housing showed a significant reduction of SIT in the second encounter with the juvenile (A2) in comparison to the first encounter (A1) (paired t-test − 30 min: t_(9)_ = 2.58, *p*<0.05; 120 min: t_(9)_ = 3.5, *p*<0.01). In contrast, socially isolated females showed no significant difference between the two encounters (Fig. 6B) (30 min: t_(9)_ = 1.02, *p*>0.05; 120 min: t_(9)_ = −0.95, *p*>0.05). Thus, social isolation impairs the SRM of females as in males. Accordingly, no significant difference was found in RDI values between males (grey bars) and females (empty bars) for both housing conditions and intervals (Fig. 6C) (t test - Grouped 30 min: t_(18)_ = 0.18, *p*>0.05; Grouped 120 min: t_(18)_ = −0.021, *p*>0.05; Isolated 30 min: t_(18)_ = −0.157, *p*>0.05; Isolated 120 min: t_(18)_ = 1.74, *p*>0.05). As previously reported [24], female rats showed less persistent investigation of the social stimuli; the SIT during their first encounter with the juvenile was significantly lower than for male rats, regardless of housing (Fig. 6D) (t-test – Grouped: t_(18)_ = −4.5, *p*<0.001; Isolated: t_(18)_ = −2.3, *p*<0.05). Thus, despite the difference in social investigation behavior between male and female rats, we did not observe any difference in their SRM or in its sensitivity to social isolation.

## Discussion

Here we have found rapid and profound but reversible effects of housing conditions on SRM in adult rats. In contrast to group-housed rats, acutely isolated rats needed a longer exposure time to acquire short-term SRM and were unable to consolidate long-term SRM. The effects of social isolation on short- and long-term SRM had different time courses and only the latter could be reversed by systemic AVP administration, indicating the involvement of different mechanisms.

The SRM paradigm was first presented 30 years ago by Thor and Holloway in a seminal study [7] that revealed the short-term nature of this memory in rats (<80 min). Several later studies reported the enhancing effect of AVP on SRM [14], [17], [22], [23]. SRM duration could also be lengthened by retroactive facilitation but not beyond a few hours [18]. Similar results were found using the closely related social discrimination paradigm [25]. It was also found, although poorly reported, that even short-term SRM acquisition needed more than 150 s of exposure time [11]. Later, the SRM paradigm has been extended to mice, where it was found to be strongly affected by housing conditions; following a single encounter, mice in group housing retained SRM for over a week, while acutely isolated mice retained the same memory for less than 60 min [13], [21]. Similarly to mice, our adult male rats acquired long-term SRM and retained it for at least a week, contrasting with many reports that adult male rats do not retain SRM for more than 1 h (reviewed in [9], [11]). Since our experiments were performed with rats in group housing, while almost all previous studies have used socially isolated rats, the different housing conditions may explain this contradiction.

Yet, our results also contradict several previous studies that did not obtain long-term SRM from rats even when kept in group housing [10], [12]. The SRM paradigm was originally designed to study differences between individuals, hence juveniles of the same strain as the adult rats were always used [8]. Here, the two juveniles used as social stimuli in each test were from two different strains, both different from the strain of the adult subjects. This methodological change most probably augmented the differences between the stimuli hence boosted SRM acquisition, allowing long-term SRM. Similarly, mice show differences in discrimination between intra- and inter-strain cues for mate recognition memory [26], and oxytocin activity is crucial for intra- but not inter-strain social recognition [27].

The acutely isolated rats did not show impaired SRM due to a behavioral change causing them to equally investigate novel and familiar juveniles. Rats which acquired SRM while in group housing investigated the familiar juvenile significantly less than a novel one even after a week in social isolation (Fig. 3B), while rats that acquired the SRM while living in isolation did not. That is, it is the impairment in consolidating long-term memory, rather than a behavioral effect of isolation, that prevented the isolated rats from discerning between the juveniles.

We showed that adult male rats, like mice [13], did not consolidate long-term SRM already after 1 day of individual housing. Moreover, as in mice [21], isolation of the rats for up to 14 days specifically impaired social recognition memory with no apparent change in object memory [28]. The rapid and specific impairment of SRM consolidation suggests that molecular processes in the neuronal network underlying social memory are strongly modulated by ongoing social activity. It should be noted that a recent study reported slight changes in myelin thickness in the medial prefrontal cortex of mice already after 2 weeks of isolation [29]. We also showed that systemic AVP injection compensated for the effect of social isolation on long-term SRM. These results may be explained if frequent social interactions are needed to maintain AVP concentrations at a sufficient level to enable consolidation of long-term SRM. Alternatively, exogenous AVP may enhance the activity in the network, thus allowing it to overcome the lack of a distinct factor caused by social isolation.

The impairments in short- and long-term SRM suggest that both SRM acquisition and consolidation are hampered by acute social isolation of adult rats. As systemic AVP administration reverses the impairment only in long-term SRM, different mechanisms appear to be responsible. This conclusion is further supported by the distinct time courses of induction and reversal displayed by the two impairments. Most notably, one day after regrouping, following a week of social isolation, the long-term SRM of adult male rats was still impaired while the short-term SRM showed a significant improvement even over that before isolation. To explain this point we hypothesize that social isolation inhibits the release, but not the synthesis, of a factor crucial for SRM acquisition. This factor, which is accumulated during the period of isolation, will be released during the next social encounter in higher than normal amounts, causing enhanced short-term SRM. In contrast, SRM consolidation to long-term memory may require other factors, the synthesis of which is inhibited by social isolation hence needs longer time to be restored.

### Conclusions

Here we showed for the first time that, if kept in group housing, both male and female adult rats can acquire long-term SRM. In contrast, acute social isolation causes a rapid impairment in both short- and long-term SRM, while object recognition memory was not affected. We found that both impairments are reversible, but with different time courses. Moreover, only the impairment in long-term SRM is reversed by systemic AVP injection, suggesting distinct mechanisms for isolation-induced impairment of short- and long-term SRM.
